# Role of GLCCI1 in inhibiting PI3K-induced NLRP3 inflammasome activation in asthma

**DOI:** 10.1016/j.pccm.2024.11.007

**Published:** 2024-12-17

**Authors:** Yingyu Zhang, Yuanyuan Jiang, Daimo Zhang, Xinyue Hu, Shuanglinzi Deng, Xiaozhao Li, Juntao Feng

**Affiliations:** a Department of Respiratory Medicine, National Key Clinical Specialty, Branch of National Clinical Research Center for Respiratory Disease, Xiangya Hospital, Central South University, Changsha, Hunan 410008, China; b Department of Clinical Laboratory, Xiangya Hospital, Central South University, Changsha, Hunan 410008, China; c National Clinical Research Center for Geriatric Disorders, Xiangya Hospital, Central South University, Changsha, Hunan 410008, China; d Department of Nephrology, Xiangya Hospital, Central South University, Changsha, Hunan 410008, China

**Keywords:** Asthma, NLRP3 inflammasome, Glucocorticoid-induced transcript 1 (GLCCI1), PI3K signaling pathway, Macrophage

## Abstract

**Background:**

Glucocorticoid-induced transcript 1 (GLCCI1) has been reported to be associated with the efficiency of inhaled glucocorticoids in patients with asthma. This study aimed to investigate the role of GLCCI1 in the regulation of nucleotide-binding oligomerization domain (NOD)-like receptor (NLR) family pyrin domain-containing 3 (NLRP3) by the phosphatidylinositol 3-kinase (PI3K) pathway in the pathogenesis of allergic asthma.

**Methods:**

The expression levels of genes encoding GLCCI1, NLRP3 inflammasome components, and PI3K pathway-related indicators were detected in cells isolated from induced sputum from patients with asthma and healthy controls. Next, we induced asthma in wild-type C57BL/6 mice and *Glcci1* knockout (*Glcci1*^-/-^) mice by injecting them with ovalbumin (OVA) and treated the asthmatic mice with a PI3K pathway inhibitor (LY294002) or left them untreated. We also performed adoptive transfer of macrophages into the mice and assessed lung inflammation, as well as GLCCI1, PI3K pathway component, and NLRP3 inflammasome component expression levels. Finally, primary bone marrow-derived macrophages (BMDMs) from wild-type and *Glcci1*^-/-^ mice were treated with OVA, either in the presence or absence of LY294002 and the NLRP3 inhibitor (MCC950), to validate our findings.

**Results:**

The mRNA level of *Glcci1* in induced sputum cells from asthmatic patients was lower compared to that of healthy controls. Additionally, *Glcci1* mRNA expression correlated negatively with NLRP3 inflammasome indicators and the PI3K pathway components, as well as with IL-1β expression in induced sputum macrophages. *In vivo, Glcci1*^-/-^ asthmatic mice showed elevated levels of airway inflammation and NLRP3 inflammasome activation compared to wild-type asthmatic mice. Surprisingly, the efficacy of LY294002 in reducing lung tissue inflammation and NLRP3 inflammasome activity in wild-type asthmatic mice was attenuated by *Glcci1* knockout. LY294002 enhanced GLCCI1 levels in macrophages within the lung tissue of wild-type asthmatic mice. Moreover, LY294002 did not inhibit lung inflammation in wild-type asthmatic mice depleted of macrophages that had received adoptive transfer of *Glcci1*^-/-^ BMDMs. *In vitro* experiments further illustrated that LY294002 suppressed NLRP3 activation by upregulating GLCCI1 expression in BMDMs. The introduction of MCC950 led to a marked decrease in NLRP3 and apoptosis-associated speck-like protein containing a caspase recruitment domain (ASC) protein levels, but did not affect the expression levels of GLCCI1 or the phospho-protein kinase B (p-AKT)/AKT ratio.

**Conclusions:**

GLCCI1 deficiency promotes asthma inflammation through PI3K-induced NLRP3 inflammasome activation.

## Introduction

Bronchial asthma is a heterogeneous condition marked by reversible airway obstruction and heightened airway reactivity,^1^ with its incidence rising year after year. Despite extensive research, the complex underlying mechanisms of asthma are still not fully understood.

The glucocorticoid-induced transcript 1 (*GLCCI1*) gene has garnered significant attention in asthma research due to its association with the glucocorticoid response, a cornerstone of asthma treatment.^2^ Specifically, Tantisira *et al*^2^ reported that a mutation leading to decreased expression of *GLCCI1* can alter the biological effects of glucocorticoids in patients with asthma. Our group also found that, in Han Chinese subjects in the mainland of China, GLCCI1 expression levels were lower in asthmatics compared with healthy individuals.^3^ Moreover, *Glcci1* knockout (*Glcci1*^-/-^) asthmatic mice exhibited more severe airway inflammation, as evidenced by increased mRNA expression levels of tumor necrosis factor-α (TNF-α), inteferon-γ (IFN-γ), and the chemokines chemokine (C-C motif) ligand (CCL) 3, CCL4, and CCL7.^4^^,^^5^ However, the specific mechanisms by which GLCCI1 dificiency exacerbates airway inflammation in asthma have not yet been fully elucidated.

Phosphatidylinositol 3-kinases (PI3Ks) are involved in airway inflammation.^6^ Administration of PI3K inhibitors has been shown to mitigate type 2 immune responses and airway hyperresponsiveness in mice with allergic asthma.^7^ The observed effects correlated with reduced expression levels of nucleotide-binding oligomerization domain (NOD)-like receptor containing pyrin domain 3 (NLRP3) inflammasome components in the primary airway epithelial cells of mice with house dust mite (HDM)-induced asthma.^8^ The NLRP3 inflammasome, an intracellular pattern recognition receptor, is composed of NLRP3, the apoptosis-associated speck-like protein containing a caspase recruitment domain (ASC), and the effector protein caspase-1, and the expression of all three is significantly increased in the lung tissues of asthmatic mice.^9^ Additionally, studies have shown that expression levels of key downstream indicators of the NLRP3 pathway (interleukin [IL]-1β and IL-18) were significantly elevated in the peripheral blood of patients with asthma.^10^ In diabetic nephropathy^11^, research has indicated that inhibition of the PI3K pathway increased GLCCI1 expression. However, there have been no reports indicating whether GLCCI1 deficiency in patients with asthma contributes to enhanced airway inflammation through activation of the PI3K pathway or activation of the NLRP3 inflammasome.

Given the established significance of these pathways in asthma-related inflammation, the aim of our study was to determine whether GLCCI1 modulates airway inflammation through its involvement in the PI3K pathway and in NLRP3 inflammasome activation. Considering that macrophages are the dominant immune cells in lung tissue and the primary source of IL-1β following NLRP3 activation,^12^ we focused on this cell type, rather than structural lung cells, in our study. By comprehensively evaluating the interplay between GLCCI1, the PI3K pathway, and NLRP3 inflammasome activation in macrophages, our study provides further insights into the molecular mechanisms underlying the pathogenesis of asthma. Our findings may help develop personalized and targeted therapies for asthma management.

## Methods

### Study design and sputum induction

A total of 48 patients diagnosed with asthma who visited the outpatient department of Respiratory and Critical Care Medicine, Xiangya Hospital, Central South University from July 22, 2021 to October 22, 2021 were recruited to participate in the study, and 15 adults who received normal physical examination results during the same period were enrolled as healthy controls. This study was approved by the Ethics Committee of Xiangya Hospital, Xiangya Medical College, Central South University (No. 201803479). The inclusion criteria for patients with asthma were as follows: diagnosis according to the guidelines for the prevention and management of bronchial asthma in China (2020 edition); age between 14 and 70 years; both male and female; no serious diseases of the brain, liver, heart, or other systemic diseases; consent to participate in the study and sign the informed consent. The exclusion criteria were: history of acute asthma attack in the past month or diagnosis of severe asthma; respiratory tract infection in the last 2 weeks; presence of chronic obstructive pulmonary disease, bronchiectasis, or other respiratory diseases; pregnancy; severe organ failure; and mental abnormalities. The inclusion criteria for the healthy control group were: no history of chronic respiratory diseases; no respiratory tract infection within the last 2 weeks; no history of atopic disease; no family history of asthma; non-smokers; and no serious systemic disease. All subjects underwent pulmonary function testing. After obtaining consent, the patients’ basic information and morbidity characteristics were recorded, and subjects meeting the inclusion criteria were enrolled in the study. Sputum induction and cell suspension preparation for reverse transcription-polymerase chain reaction (RT-PCR) and flow cytometry were performed as described previously.^13^

### Animals and ovalbumin (OVA)-induced asthma model

All experimental procedures were performed in accordance with the Chinese Council of Animal Care Guidelines and approved by the Central South University Animal Care Committee (No. 201803478). Sex-matched wild-type (WT) and *Glcci1* gene knockout (*Glcci1*^-/-^) C57BL/6 mice (6–8 weeks) were raised in the Experimental Animal Center of Central South University in Changsha, Hunan Province under specific pathogen free (SPF) conditions. The homozygous *Glcci1*^-/-^ mouse line was constructed as previously described,^4^ and the GLCCI1 deficiency in the KO mice was validated (Supplementary Fig. 1). In brief, mice were injected intraperitoneally (i.p.) on days 0 and 14 with OVA (75 µg, Sigma, Saint Louis, Missouri, USA) and aluminum hydroxide (2 mg). From days 21 to 23, all sensitized mice were exposed to atomized 5% OVA for 30 minutes per day. Mice in the vehicle group received sterile saline for pseudo-sensitization and challenge. Mice in the LY294002+OVA group were intraperitoneally injected with LY294002 (80 mg/kg) daily, 30 minutes before exposure to the OVA aerosol. The vehicle group was treated with dimethyl sulfoxide (DMSO), the LY294002 solvent. All mice were sacrificed on day 24, and samples were collected for further study.

### Cell culture

Primary bone marrow derived macrophages (BMDMs) were obtained from mice as previously described.^14^ Briefly, bone marrow was collected, the red blood cells were lysed, and the remaining cells were suspended in Roswell Park Memorial Institute (RPMI) 1640 culture medium containing 10% fetal bovine serum, penicillin/streptomycin, and 40 ng/ml macrophage colony-stimulating factor. The cells were then seeded into tissue culture dishes of appropriate size and maintained at 37°C, with the culture medium changed every 2 days. The cells were ready for use in experiments on day 7. For pharmacological treatment, the PI3K inhibitor LY294002, PI3K inhibitor wortmannin, NLRP3 inhibitor MCC950 were purchased from MedChem Express (Monmouth Junction, NJ, USA) and used to treat BMDMs according to the experimental design. For macrophages activation, endotoxin-free OVA (Hyglos, Bernried am Starnberger See, Bavaria, Germany) was added at a concentration of 100 µg/ml. BEAS-2B cells and A549 cells were obtained from the National Clinical Research Center for Respiratory Diseases subcenter (Beijing, China). All cells were incubated at 37°C with 5% CO_2_.

### Measurement of bronchial responsiveness

Mouse airway responsiveness to methacholine was measured within 24 h after the final challenge using whole-body plethysmography (Buxco Electronics, Wilmington, NC, USA). Mice were exposed to aerosolized methacholine solutions at different concentrations (0, 6.0, 12.5, 25.0, and 50.0 mg/ml in normal saline) for 1 minute. Lung resistance (RL) was then measured.^4^^,^^5^ The results are expressed as a percentage of the baseline RL values recorded after saline exposure for each methacholine concentration.

### Macrophage depletion and macrophage adoptive transfer studies

To deplete macrophages, C57BL/6 mice were treated with liposomal clodronate (FormMax Scientific, Waltham, MA, USA) or empty liposomes (Control). According to a previously published protocol, each mouse was exposed to liposomal clodronate (200 µL/mouse daily) for 2 consecutive days prior to each OVA challenge.^15^ Additionally, BMDMs (1 × 10^6^/mouse) from mice with different genotypes were injected into the tail vein 24 h after treatment with liposomal clodronate. Three hours after adoptive transfer, the mice were subjected to aerosol inhalation of OVA and LY294002. Vital signs and body weight were closely monitored, and samples were collected on day 24.

### Flow cytometry analysis

For analysis of bronchoalveolar lavage fluids and cell type percentages, eosinophils were characterized as Siglec-F^+^CD11c^-^, alveolar macrophages as Siglec-F^+^CD11c^+^, granulocytes as lymphocyte antigen 6 complex locus G (Ly6G)^+^CD11b^+^CD11c^-^, and T lymphocytes as CD3^+^CD11c^-^. Macrophages in lung tissue samples were detected by staining with anti-mouse CD45^-^ allophycocyanin-cyanine 7 (APC-Cy7) and F4/80^-^ phycoerythrin-cyanine 7 (PE-Cy7). To analyze IL-1β expression in macrophages isolated from induced sputum, the cells were stained with anti-human CD45-APC, CD68-PE, and IL-1β-BV421. All samples were analyzed using a DxP Athena™ Flow Cytometer (Cytek, Fremont, CA, USA), and the data were processed with FlowJo software (FlowJo, Ashland, OR, USA).

### Histopathological analysis

The left lung was fixed in 4% paraformaldehyde for 48 h before being embedded in paraffin and processed according to routine protocols. Serial 3-µm tissue sections were stained with hematoxylin and eosin (H&E) and Periodic Acid-Schiff (PAS). The stained sections were then observed under a light microscope to detect morphological changes in the lung. For immunostaining, the lung section was probed with first antibody (GLCCI1, F4/80) and then stained with a fluorescent secondary antibody of the corresponding species (Servicebio, Wuhan, China). A total of six mice from each group were analyzed. Histopathology and peribronchial and perivascular inflammation were evaluated and graded.^16^

### Western blot analysis

Lung tissues and cultured cells were homogenized in radio immunoprecipitation assay (RIPA) lysis buffer (Biyuntian, Shanghai, China). The proteins were then subjected to western blotting using established techniques. Membranes were blocked with 5% nonfat dry milk or 5% bovine serum albumin (for phosphorylated proteins) dissolved in Tris-buffered saline with Tween-20 (TBST) for 1 hour and then incubated at 4°C overnight with primary antibodies against NLRP3 (1:1000, Danvers, MA, USA), ASC (1:1000, Cell Signaling Technology [CST], Danvers, Massachusetts, USA), pro-caspase-1 and cleaved-caspase-1 (1:1000, Immunoway, Plano, TX, USA), IL-1β (1:1000, R&D, Minneapolis, MN,USA), IL-18 (1:1000, ZEN BIO, Chengdu, Sichuan, China), GLCCI1 (1:1000, Abclonal, Beijing, China), phospho-protein kinase B (p-AKT) (1:1000, CST), AKT (1:1000, CST), and glyceraldehyde-3-phosphate dehydrogenase (GAPDH) (1:5000, Proteintech, Wuhan, Hubei China). The membranes were then incubated with horseradish peroxidase–conjugated secondary antibodies for 1 hour at room temperature. The results were expressed as the ratio of the mean band density for the experimental groups to that for the control group, normalized to glyceraldehyde-3-phosphate dehydrogenase (GAPDH) as an internal control. Blots were visualized using an enhanced chemiluminescence detection system (Syngene, Cambridge, MA, USA).

### Real-time quantitative polymerase chain reaction (Real-time qPCR)

Quantitative RT-PCR analysis was performed using SYBR Premix Ex Taq (Takara, Dalian, China), as previously described.^4^ The relative expression of each target gene was normalized to β-actin expression. Primers specific for GAPDH, GLCCI1, NLRP3, ASC, caspase-1, IL-1β, IL-18, PI3K-α, PI3K-β, PI3K-γ, and PI3K-δ were designed using Primer Premier 5.0 (Ottawa, ON, Canada) and produced by Sangon Biotech (Shanghai, China). Their sequences are listed in Supplementary Table 1. For samples without a naive control, gene expression was calculated using the 2^-ΔCt^ method, with *GAPDH* as the internal reference. Other samples were calculated with 2^-ΔΔCt^ method, with *GAPDH* as the internal reference.

### Inflammatory cytokines detection

The IL-4, IL-5, IL-13, TNF-α, and IL-1β levels were measured using enzyme-linked immunosorbent assay (ELISA) kits (RayBiotech, Norcross, GA, USA) based on the manufacturer's instructions.

### Statistical analysis

All normally distributed data are expressed as mean ± standard error of the mean (SEM), and non-normally distributed data are shown as median (Q_1_, Q_3_). For normally distributed data with homogeneous variance, two groups were compared by independent samples *t*-test, multiple groups were compared by one-way analysis of variance ANOVA, multiple groups with two factors were compared by two-way ANOVA with Sidak’s multiple comparison test, and correlation analysis was performed using the Pearson correlation coefficient. For samples that did not follow a normal distribution or had uneven variance, the Mann-Whitney *U* test was used for comparisons between two groups, the Dunnett's test for comparisons between multiple groups, and the Kruskal-Wallis *H* test for comparisons between multiple groups with two factors. Spearman correlation coefficient was used for correlation analysis. GraphPad Prism 8.0 software (Bethesda, MD, USA) was used to perform all statistical analyses, and *P* < 0.05 was considered statistically significant.

## Results

### Correlation analysis of NLRP3 inflammasome component, PI3K pathway component, and GLCCI1 expression levels in induced sputum from patients with asthma

**Fig. 1:**
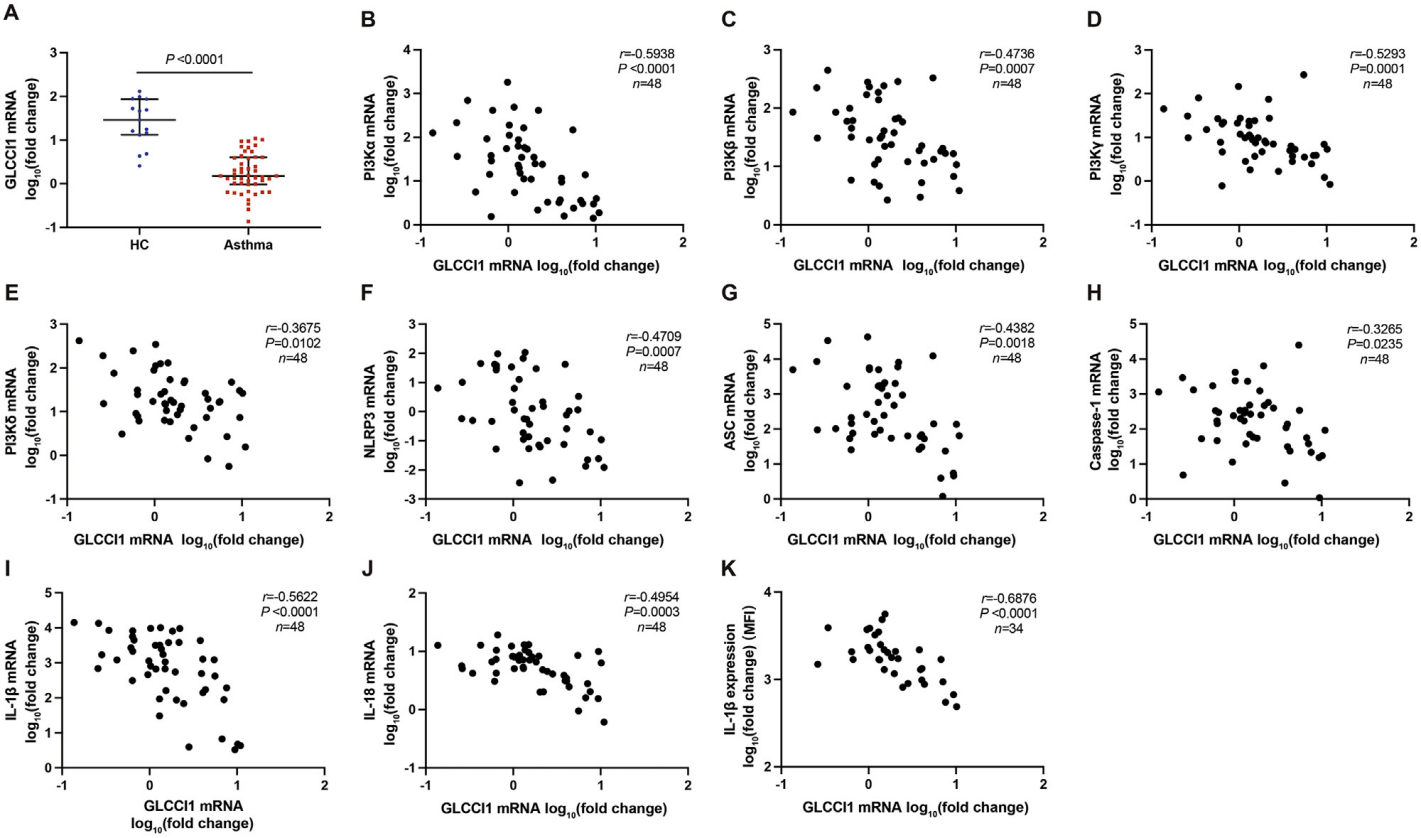
(A) Expression of *Glcci1* in induced sputum cells of each group was detected by RT-PCR in asthma group (*n* = 48) and healthy control group (HC, *n* = 15). RT-PCR was used to detect PI3K subunit components (B) PI3K-α, (C) PI3K-β, (D) PI3K-γ, (E) PI3K-δ, and NLRP3 inflammasome components (F) NLRP3, (G) ASC, (H) caspase-1, (I) IL-1β, (J) IL-18 in induced sputum cells of asthma group (*n* = 48). Further correlation analysis was carried out between GLCCI1 and these indicators. (K) Expression of IL-1β in CD68^+^ macrophages induced by asthma patients as assessed by flow cytometry. Flow Jo (Flow Jo, Ashland, Oregon, USA) was used to analyze the MFI of IL-1β, and the correlation analysis was performed with *Glcci1* gene level (*n* = 34). Error bars represent medians (Q_1_, Q_3_) (A). Spearman's correlation (B–K) and Mann–Whitney *U* test (A) were used. ASC: Apoptosis- associated speck like protein containing a caspase recruitment domain; GAPDH: Glyceraldehyde-3-phosphate dehydrogenase; GLCCI1: Glucocorticoid-induced transcript 1; IL: Interleukin; MFI: Mean fluorescence intensity; mRNA: Messenger RNA; NLRP3: Nod-like receptor containing pyrin domain 3; PI3K: Phosphatidylinositol 3-kinase; RT-PCR: Reverse transcription-polymerase chain reaction.

Our findings revealed a significant decrease in *Glcci1* mRNA levels in induced sputum from patients with asthma compared with healthy controls (Fig. 1A). We then explored the relationship between GLCCI1 and both the PI3K pathway and NLRP3 inflammation in patients with asthma. Fig. 1B–E illustrate a negative correlation between GLCCI1 expression and the expression levels of four PI3K subunits (*P* < 0.05). Additionally, a negative correlation between GLCCI1 expression and NLRP3, ASC, caspase-1, IL-1β, and IL-18 expression levels was observed (each *P* < 0.05, Fig. 1F–J). Using flow cytometry, we assessed IL-1β protein levels in induced sputum macrophages from patients with asthma and observed a negative correlation between IL-1β protein levels and *Glcci1* gene expression (*P* < 0.05, Fig. 1K).

### PI3K inhibitors reduce airway inflammation and NLRP3 inflammasome activity while increasing GLCCI1 expression in an OVA-induced mouse model of asthma

**Fig. 2:**
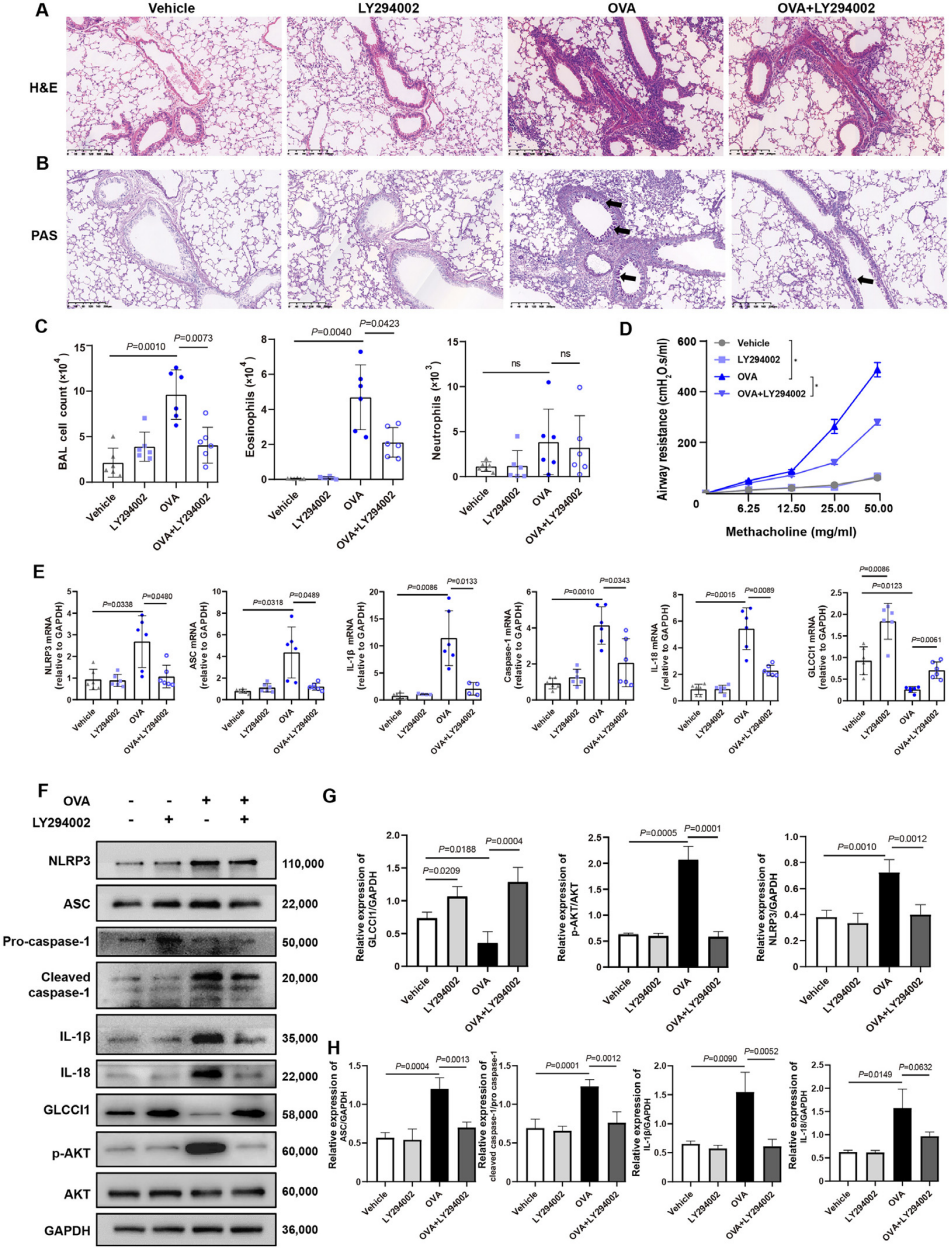
LY294002 reduced airway inflammation, NLRP3 inflammasome activity, and increased GLCCI1 expression in OVA-induced asthmatic mice. (A) Representative H&E staining images (original magnification × 100). (B) Representative PAS staining images (original magnification × 100). (C) BAL fluid total and differential (eosinophils, neutrophils) cell counts as assessed by flow cytometry. (D) AHR in terms of RL to increasing doses of MCh (*n* = 6). (E) Expression of NLRP3 inflammasome components in OVA-induced asthmatic mice with or without LY294002 as detected by RT-PCR (*n* = 6). (F) Western blot analysis of NLRP3 inflammasome components, GLCCI1, PI3K pathway components in the lung tissues of OVA-induced asthmatic mice with or without LY294002. (G, H) Quantitative analysis of western blots (*n* = 5). Data are presented as means ± SEMs. Dunnett's test (C, E, G, H) and Sidak's test (D) were used. *P <0.001. AHR: Airway hyperresponsiveness; ASC: Apoptosis-associated speck like protein containing a caspase recruitment domain; BAL: Bronchoalveolar lavage; GAPDH: Glyceraldehyde-3-phosphate dehydrogenase; GLCCI1: Glucocorticoid-induced transcript 1; MCh: Methacholine; mRNA: Messenger RNA; H&E: Hematoxylin and eosin; IL: Interleukin; LY294002: PI3K pathway inhibitor; NLRP3: NOD-like receptor containing pyrin domain 3; ns: Not significant; OVA: Ovalbumin; PAS: Periodic Acid-Schiff; PI3K: Phosphoinositide 3-kinase; RL: Lung resistance; RT-PCR: Reverse transcription-polymerase chain reaction; p-AKT: Phospho-protein kinase B (AKT); SEM: Standard error of the mean.

Next, a PI3K pathway inhibitor (LY294002) was applied to an OVA-induced mouse model of asthma. We found that LY294002 significantly inhibited pulmonary inflammation, as illustrated by decreased infiltration of inflammatory cells in the lung tissue and reduced eosinophil infiltration in the alveolar cavities of asthmatic mice (Fig. 2A–C, *P* < 0.05). As expected, a reduction in airway hyperresponsiveness was also observed (Fig. 2D, *P* < 0.05). This trend was further validated by a reduction in the expression of NLRP3 inflammasome components at both the gene and protein expression level after LY294002 administration. This was associated with increased expression of GLCCI1 at the gene and protein level in asthmatic mice (Fig. 2 E–H, *P*<0.05).

### GLCCI1 is involved in the PI3K pathway activation in asthma

**Fig. 3:**
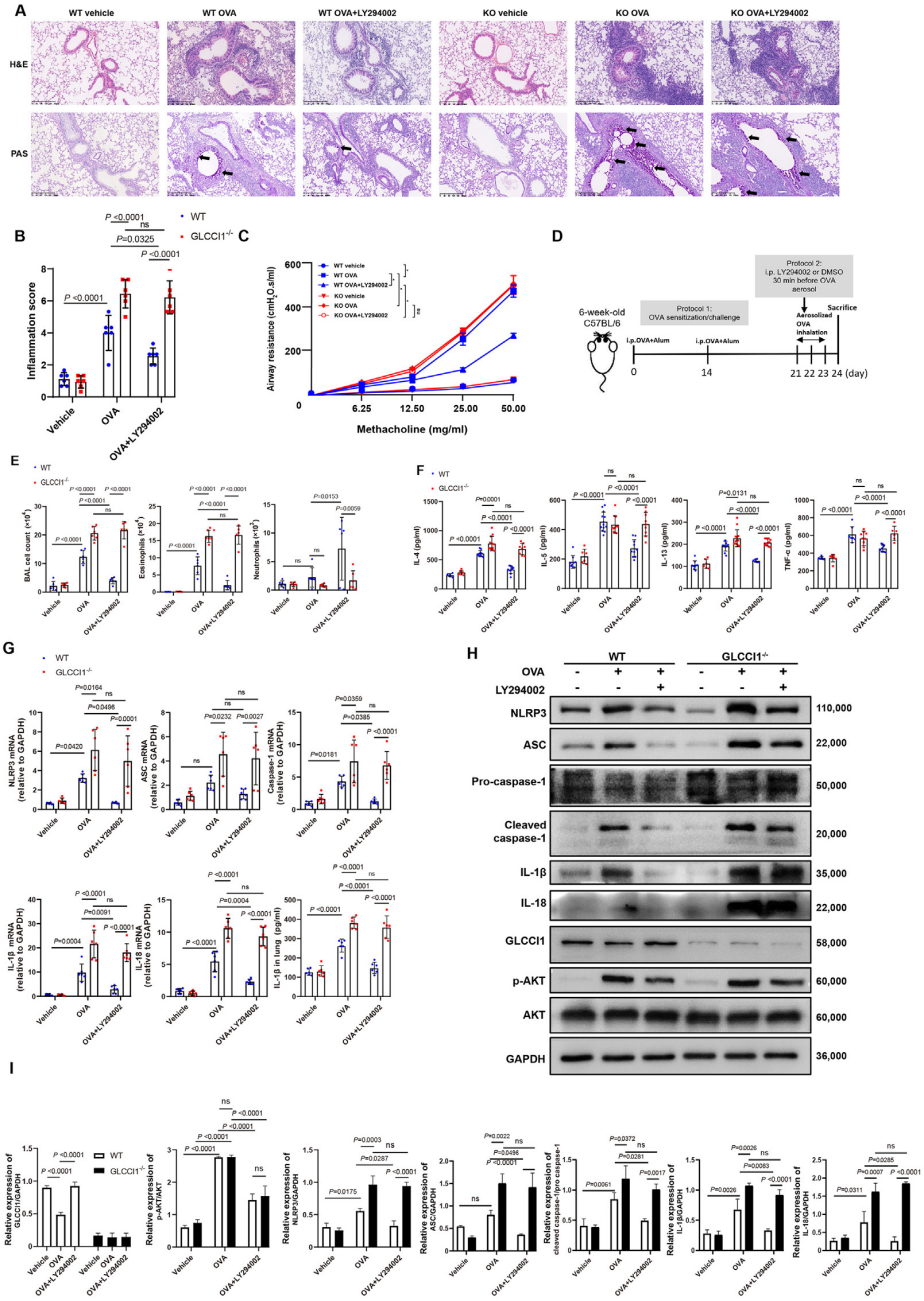
GLCCI1 is involved in the PI3K pathway activation in asthma. (A) Representative H&E staining images (original magnification × 100) and PAS staining images (original magnification × 100). (B) Inflammation score (*n* = 6). (C) AHR in terms of RL to increasing doses of MCh (*n* = 6). (D) Experimental protocol of the study. (E) BALF total and differential (eosinophil, neutrophil) cell counts as assessed by flow cytometry (*n* = 6). (F) Levels of cytokines in the lung tissue protein of mice in each group were detected by ELISA (*n* = 6). (G) RT-PCR and ELISA were used to detect the expression levels of NLRP3 inflammasome components and activated protein in lung tissues of OVA-induced asthma WT and GLCCI1^-/-^ mice (*n* = 6) treated with or without LY294002. (H, I) Western blot analysis of NLRP3 inflammasome components and activated protein, GLCCI1, and PI3K pathway components in the lung tissues of mice in each group (H) and their protein quantitative analysis (I) (*n* = 5). Data are presented as means ± SEMs. Two-way ANOVA with Sidak's multiple comparison test was used (B, C, E, F, G, I). *P <0.001. AHR: Airway hyperresponsiveness; ASC: Apoptosis-associated speck like protein containing a caspase recruitment domain; BAL: Bronchoalveolar lavage; BALF: Bronchoalveolar lavage fluid; DMSO: Dimethyl sulfoxide; ELISA: Enzyme-linked immunosorbent assay; GAPDH: Glyceraldehyde-3-phosphate dehydrogenase; GLCCI1: Glucocorticoid-induced transcript 1; IL: Interleukin; i.p.: Intraperitoneally; KO: Knock-out; LY294002: PI3K pathway inhibitor; MCh: Methacholine; mRNA: Messenger RNA; H&E: Hematoxylin and eosin; NLRP3: NOD-like receptor containing pyrin domain 3; ns: Not significant; OVA: Ovalbumin; p-AKT: Phospho-protein kinase B (AKT); PAS: Periodic Acid-Schiff; PI3K: Phosphoinositide 3-kinase; RL: Lung resistance; RT-PCR: Reverse transcription-polymerase chain reaction; SEM: Standard error of the mean; TNF-α: Tumor necrosis factor-α; WT: Wild-type.

To investigate the role of GLCCI1 in regulating the PI3K pathway, we generated an OVA-induced asthma model using both WT and *Glcci1*^-/-^ mice and evaluated airway inflammation following treatment with LY294002 (Fig. 3A–E). In *Glcci1*^-/-^ asthmatic mice, we observed significantly increased infiltration of inflammatory cells, goblet cell metaplasia, and mucus secretion in the lung tissues compared with WT asthmatic mice (Fig. 3A–B). Similarly, *Glcci1*^-/-^ asthmatic mice exhibited more severe airway resistance that was not alleviated by LY294002 treatment (Fig. 3C). Flow cytometry analysis of immune cells in bronchoalveolar lavage fluid (BALF) showed that the total number of airway cells and eosinophils was significantly higher than that in the control group, but that LY294002 failed to reverse this effect in *Glcci1*^-/-^ asthmatic mice as it did in wild-type asthmatic mice (Fig. 3E). Similarly, higher IL-4, IL-5, IL-13 and TNF-α levels were observed in the lung tissue of *Glcci1*^-/-^ asthmatic mice compared with WT mice. LY294002 treatment did not mitigate the increase in inflammation in *Glcci1*^-/-^ asthmatic mice (Fig. 3E–F, *P* < 0.05). We also found NLRP3 inflammasome activation was significantly higher in *Glcci1*^-/-^ asthmatic mice, as indicated by increased levels of cleaved caspase-1, IL-1β, and IL-18 (Fig. 3G–I). However, GLCCI1 deficiency did not affect AKT phosphorylation levels in the lung tissue of asthmatic mice (Fig. 3H). Additionally, treatment with PI3K pathway inhibitors did not reduce the elevated NLRP3 activity observed in *Glcci1*^-/-^ asthmatic mice (Fig. 3H–I).

### PI3K regulates GLCCI1 expression in macrophages as part of the pathogenesis of asthma

**Fig. 4:**
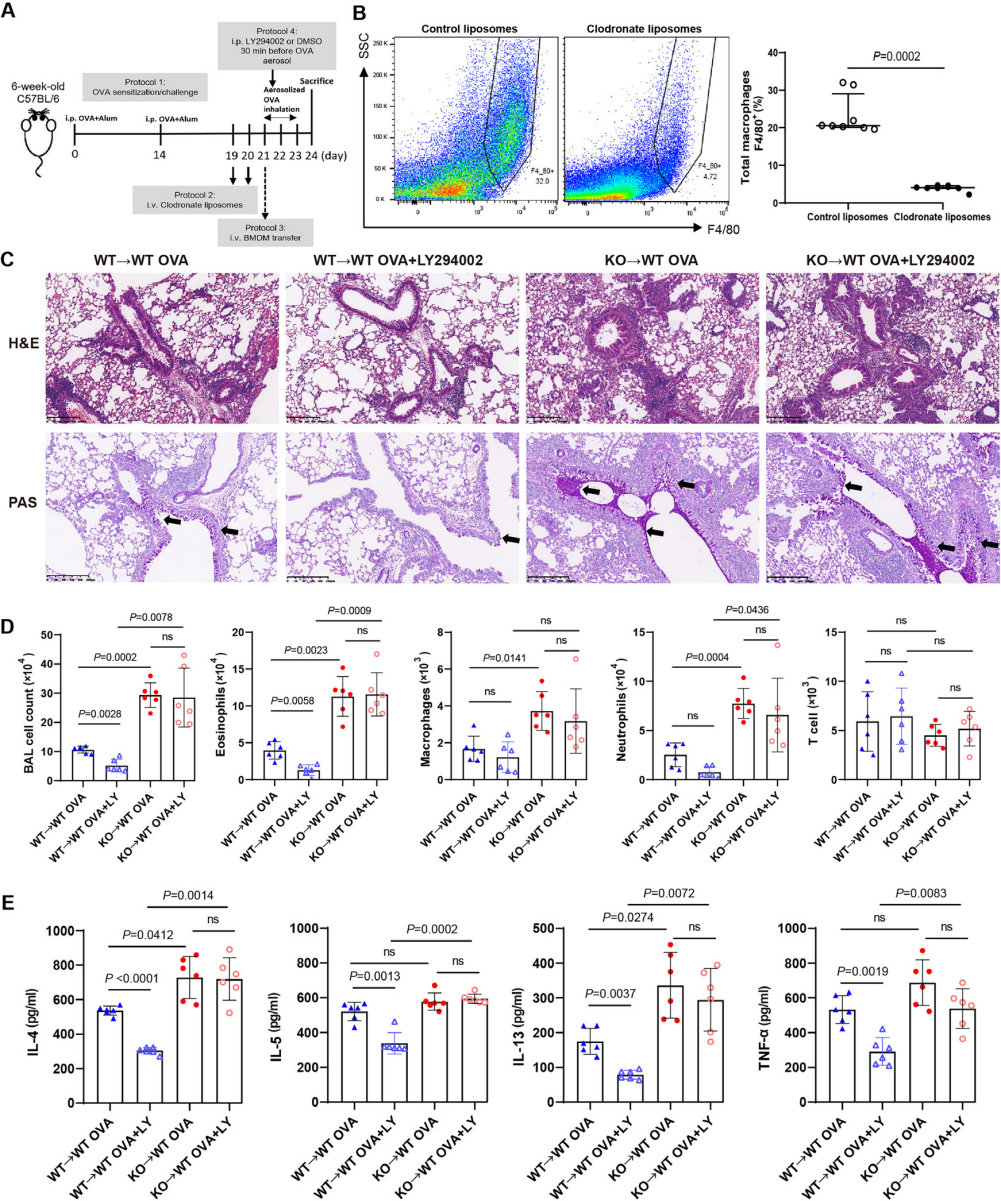
LY294002 reduces airway inflammation in asthma by increasing macrophage GLCCI1 expression. (A) Experimental protocol of macrophage clearance and adoptive transefer. (B) Representative gaiting strategy of total macrophages (left) from the lung single-cell suspensions in both control liposomes-treated mice and clodronate liposomes-treated mice. Comparisons of total macrophage frequencies (percentage of CD45^+^ cells) between the control group and the experimental group (right) was performed, *n* = 8. (C) Representative H&E staining images (original magnification × 100) and PAS staining images (original magnification × 100). (D) BALF total and differential (eosinophil, macrophage, neutrophil, and T lymphocyte) cell counts were assessed by flow cytometry (*n* = 6). (E) Levels of cytokines in lung tissues of mice in each group were detected by ELISA (*n* = 6). Data are presented as medians (Q_1_, Q_3_) (B) or means ± SEMs (D, E). Mann-Whitney *U* test (B) and Dunnett's test (D, E) were used. BAL: Bronchoalveolar lavage; BMDM: Bone marrow-derived macrophages; DMSO: Dimethyl sulfoxide; ELISA: Enzyme-linked immunosorbent assay; GLCCI1: Glucocorticoid-induced transcript 1; H&E: Hematoxylin and eosin; IL: Interleukin; i.p.: Intraperitoneally; i.v.: Intravenously; KO: Knock-out; LY294002: PI3K pathway inhibitor; ns: Not significant; OVA: Ovalbumin; PAS: Periodic Acid-Schiff; SEM: Standard error of mean; SSC: Sider scatter; TNF-α: Tumor necrosis factor-α; WT: Wild-type.

In order to explore whether GLCCI1 affects macrophages in the context of asthma, we performed macrophage depletion and adoptive transfer experiments (Fig. 4A). As shown in Fig. 4B, clodronate liposomes achieved over 80% macrophage clearance efficiency. Furthermore, in asthmatic mice that received WT macrophages (WT→WT), LY294002 treatment significantly reduced eosinophil infiltration in the airway submucosa, airway, and perivascular regions (Fig. 4C). There was also a reduction in goblet cell number and mucus hypersecretion (arrow), and the total number of cells and eosinophils in the BALF decreased significantly (Fig. 4D). Additionally, inflammatory factor secretion in the lung tissue was reduced (Fig. 4E). In contrast, asthmatic mice that received adoptive transfer of *Glcci1*^-/-^ macrophages (KO→WT) displayed significantly thickened airway walls, increased eosinophil infiltration, a higher number and volume of goblet cells, and mucus plug formation in some airways (arrow). These mice also exhibited elevated numbers of total cells, eosinophils, macrophages, and neutrophils in the BALF, and these effects were not reversed by treatment with LY294002. In addition, a markedly higher level of inflammatory cytokine secretion in the lung tissue was observed that was not significantly reduced following LY294002 treatment (Fig. 4E).

### The PI3K–GLCCI1–NLRP3 signaling cascade in macrophages plays a role in asthma pathogenesis

**Fig. 5:**
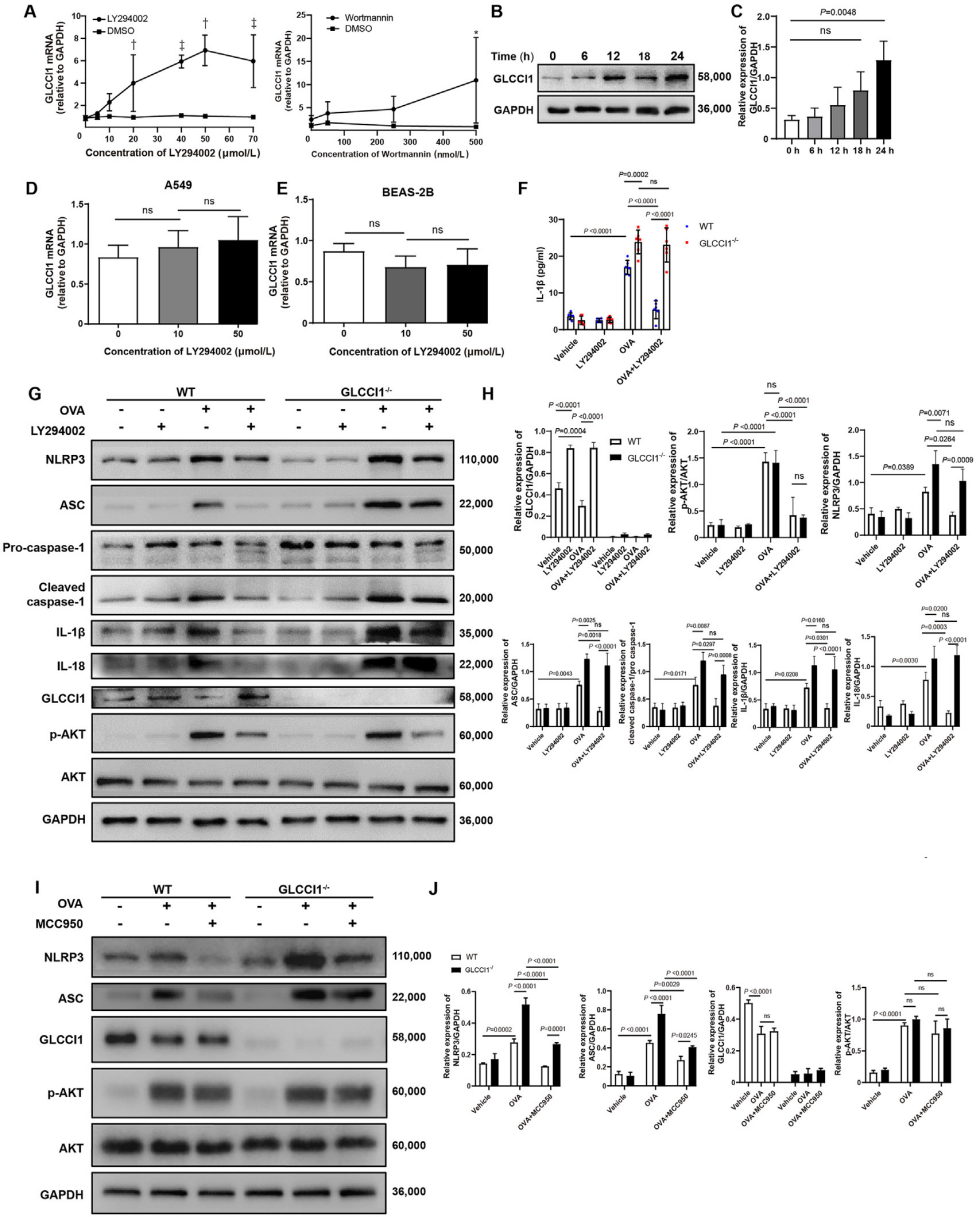
PI3K–GLCCI1–NLRP3 signaling cascade in OVA-activated macrophages. (A) Different concentrations of LY294002 or wortmannin stimulated BMDM for 24 h, and the corresponding concentration of DMSO was used as the control, the mRNA levels of *Glcci1* in each group were detected by RT-PCR, and repeated independently for three times. (B) LY294002 at 40 µmol/L concentration was used to stimulate BMDM for different lengths of time, and the protein levels of GLCCI1 in each group were detected by western blot. (C) Quantitative analysis of western blots was performed. Different concentrations of LY294002 stimulated A549 (D) and BEAS-2B (E) cells for 24 h. (F–H) BMDMs were stimulated with 100 µg/mL OVA for 24 h with or without LY294002 (40 µmol/L). IL-1β released into culture supernatants was measured by ELISA (F). Protein levels (G, H) of NLRP3 inflammasome components, GLCCI1, PI3K pathway in the BMDMs from different groups were shown. (I, J) BMDMs were stimulated with 100 µg/mL OVA for 24 h with or without MCC950 (100 µmol/L). Representative images (I) and relative protein levels (J) of NLRP3, ASC, GLCCI1, PI3K pathway components in the BMDMs from different groups are shown. Data are presented as means ± SEMs. For multiple comparisons, *P* values were determined by two-way ANOVA with Sidak' s test (A, F, H, J), or one-way ANOVA with Dunnett's test (C, D, E). *P <0.05, ^†^*P* <0.01, ^‡^*P* <0.001. ASC: Apoptosis-associated speck-like protein containing a caspase recruitment domain; BMDM: Bone marrow-derived macrophage; DMSO: Dimethyl sulfoxide; ELISA: Enzyme-linked immunosorbent assay; GAPDH: Glyceraldehyde-3-phosphate dehydrogenase; GLCCI1: Glucocorticoid-induced transcript 1; IL: Interleukin; LY294002: PI3K pathway inhibitor; MCC950: NLRP3 inhibitor; mRNA: Messenger RNA; NLRP3: Nod-like receptor containing pyrin domain 3; ns: Not significant; OVA: Ovalbumin; p-AKT: Phospho-protein kinase B (AKT); PI3K: Phosphatidylinositol 3-kinase; RT-PCR: Reverse transcription-polymerase chain reaction; SEM: Standard error of the mean; WT: Wild-type.

Next, we conducted *in vitro* experiments using bone marrow-derived macrophages (BMDMs) to validate our *in vivo* findings from the mouse model of asthma. We treated BMDMs with various concentrations of LY294002 and wortmannin, another common PI3K pathway inhibitor. However, LY294002, even at lower concentrations, led to a more pronounced increase in GLCCI1 expression compared with wortmannin (Fig. 5A). Additionally, we observed a significant increase in GLCCI1 protein expression by BMDMs 24 hours after treatment with LY294002 (Fig. 5B–C). Based on these findings, we treated cells with 40 µmol/L LY294002 for 24 hours in two epithelial cell lines, A549 and BEAS-2B, however, no differences were observed (Fig. 5D–E).

We then applied LY294002 to BMDMs treated with OVA. We found that NLRP3, ASC, and cleaved caspase-1 (a downstream target of NLRP3) expression levels in BMDMs from *Glcci1*^-/-^ mice were significantly higher than in BMDMs from WT mice. Levels of IL-1β, another molecule downstream of NLRP3, were higher in both cell supernatants (Fig. 5F) and *Glcci1*^-/-^ mouse BMDMs (Fig. 5G-H). Applying PI3K pathway inhibitors resulted in no statistically significant change in NLRP3 activity in *Glcci1*^-/-^ BMDMs; indeed, NLRP3 activity in these cells remained notably high compared with that in WT BMDMs. This indicates that the inhibitory effect of LY294002 on NLRP3 is significantly impaired in the absence of GLCCI1 in macrophages. As shown in Fig. 5G-H, there was no significant difference in the ratio of p-AKT/AKT, an indicator of PI3K pathway activation, between the WT and *Glcci1*^-/-^ groups.

We also conducted inhibitory experiments targeting NLRP3 using MCC950, a specific NLRP3 inhibitor. The introduction of MCC950 led to a marked decrease in NLRP3 and ASC protein levels in both wild-type (WT) and *Glcci1*-deficient (*Glcci1*^-^/^-^) OVA-treated groups, as depicted in Fig. 5I–J. However, the inhibition of this molecule did not affect the expression levels of GLCCI1 or the p-AKT/AKT ratio (Fig. 5I–J). This suggests a unidirectional regulatory effect of GLCCI1 on NLRP3, rather than a reciprocal influence. In essence, our findings point to the existence of a PI3K–GLCCI1–NLRP3 signaling pathway that is activated in macrophages in response to OVA stimulation.

## Discussion

The *GLCCI1* rs37973 mutation inhibits GLCCI1 transcription in non-Hispanic Caucasian patients with asthma, which is related to inhaled corticosteroid (ICS) efficacy.^2^ In addition, this *GLCCI1* mutation has a similar effect on ICS efficacy in Tunisian adults, Japanese adults, and Chinese adults and children with asthma.^17^, ^18^, ^19^ However, the mechanisms underlying this effect remain unknown. In our previous study, we found that GLCCI1 expression levels are related to the risk of asthma, with GLCCI1 acting as a protective factor against the condition. Moreover, we recently reported decreased methylation of *GLCCI1* in peripheral blood mononuclear cells from patients with asthma and showed that multiple *GLCCI1* CpG methylation sites are positively correlated with GLCCI1 expression.^19^ These findings suggest that GLCCI1 plays an important role in asthma pathogenesis.

PI3K is a crucial intracellular signal-transduction molecule. Class I PI3Ks, which are the most well-studied, are heterodimers composed of catalytic and regulatory subunits. The four catalytic subunits are p110α, p110β, p110δ, and p110γ. Among these, p110α and p110β are broadly involved in embryonic development and cell proliferation, while p110δ and p110γ are predominantly expressed in immune cells. This distribution suggests that p110δ and p110γ play vital roles in both the innate and adaptive immune systems, making them potential targets for PI3K-related drug development.^20^, ^21^, ^22^ PI3K-α, PI3K-γ, and PI3K-δ have been reported to be involved in lung inflammation in conditions such as asthma and COPD.^23^^,^^24^ In patients with COPD, elevated PI3Kα activity increases susceptibility to influenza.^25^ PI3Kγ is associated with the secretion of inflammatory cell mediators in asthma, such as macrophage migration inhibitory factor (MIF)^26^ and IL-13^27^. PI3Kγ can also promote eosinophil chemotaxis and degranulation.^28^ PI3Kδ is associated with a variety of effector cell types involved in the Th2 immune response, mainly eosinophils. Specifically, PI3Kδ promotes the growth, differentiation, adhesion, and transport of eosinophils by promoting the expression of IL-5, CCL11, CCL5, and other molecules.^29^ Relevant studies on the use of PI3K inhibitors in asthma have also made important progress. In animal models of asthma, wortmannin and LY294002, PI3K/AKT inhibitors with low selectivity, significantly reduced the type 2 immune response and airway hyperreactivity. In addition, the selective PI3Kδ inhibitor IC87114 significantly inhibited airway hyperreactivity, eosinophil airway inflammation, and mitochondrial reactive oxygen species (mtROS) production in a fungus-induced mouse model of allergic airway inflammation.^30^, ^31^, ^32^

We previously reported that *GLCCI1* knockout significantly influenced 82.1% of genes encoding components involved in PI3K pathway.^20^ A recent study analyzing gene and microRNA expression levels in peripheral blood T cells from patients with severe asthma treated with benralizumab showed a correlation between the PI3K pathway and GLCCI1. These findings suggest that GLCCI1 may be a downstream gene of PI3K, although further verification is needed.^33^ Another study reported that the PI3K pathway can regulate the development of diabetic nephropathy by modulating GLCCI1 expression in glomerular podocyte foot processes.^11^ Therefore, it is necessary to further explore the relationship between GLCCI1 and the PI3K pathway in asthma.

The NLRP3 inflammasome is a critical mediator of the innate immune response and has been associated with the development of asthma. Some studies have proposed that NLRP3 is a transcriptional regulatory molecule necessary for Th2 cell differentiation^34^ and that the NLRP3 inflammasome can promote the macrophage M2 polarization and participate in asthma pathogenesis.^35^ Both clinical and experimental studies have shown that NLRP3 is present at high levels in the sputum and peripheral blood of patients’ asthma and in OVA-allergic airway inflammation (AAI) models.^36^ Another study reported that PI3K inhibition can reduce NLRP3 inflammasome activation in primary airway epithelial cells collected from an HDM-induced mouse model of asthma.^11^ PI3Kδ, as an important regulator of mtROS, can regulate NLRP3 inflammasome activity through mtROS in the airway epithelium.^36^ In this study, we found that treating OVA-AAI mice with LY294002 significantly inhibited NLRP3 inflammasome activity in lung tissue, which is consistent with the PI3K–NLRP3 chain of action reported in asthma.^11^ We previously found that weakening GLCCI1 expression increased p38 MAPK phosphorylation.^4^ In the present study, we showed that a PI3K inhibitor regulated GLCCI1 expression both *in vivo* and *in vitro*. The NLRP3 inflammasome acts as the downstream effector of the PI3K pathway and the p38 MAPK pathway.^11^^,^^37^ Here we found that GLCCI1 expression was negatively correlated with the expression of NLRP3 inflammasome-related mRNAs (NLRP3, ASC, caspase-1, IL-1β, and IL-18) to varying degrees in clinical samples. However, the effect of a PI3K inhibitor on the NLRP3 inflammasome was significantly abrogated in *Glcci1*^-/-^ asthmatic mice, suggesting that PI3K may regulate NLRP3 through GLCCI1.

When harmful stimuli trigger a response in the airways, the innate immune system initiates a cascade of signals through pattern recognition receptors (PRRs). NLRP3 is a key intracellular PRR that plays a pivotal role in this process.^10^ As the first cell type responding to PRR activation, macrophages showed a heightened activation of the NLRP3 inflammasome.^38^ There is a study showing that mice infected with rhinovirus had an increased expression of NLRP3 and IL-1β in subepithelial macrophages, whereas their expression levels in epithelial cells were comparatively low.^39^ Furthermore, IL-1β expression at the gene and protein level was reduced in the lungs of these infected mice following macrophage depletion, emphasizing the robust inflammatory cytokine secretion capability of macrophages in inflammation.^16^

In this study, we observed an inverse relationship between IL-1β levels and *GLCCI1* mRNA levels in sputum from patients with asthma. Treatment with LY294002, a PI3K inhibitor, has been shown to significantly enhance GLCCI1 expression in macrophages in the lung tissue of asthmatic mice. In our adoptive immunity studies, we transferred both wild-type and *GLCCI1*-deficieny bone marrow–derived macrophages (BMDMs) into asthmatic mice depleted of their endogenous macrophages. We found that, in the absence of GLCCI1, LY294002 was less effective in reducing airway inflammation. This indicates that GLCCI1 expression in macrophages is crucial for enabling PI3K inhibitors to decrease asthma symptoms. Recent studies have found that exposing alveolar macrophages to ovalbumin increases NLRP3 and IL-1β production.^40^ An deficiency of GLCCI1 in macrophages significantly reduced the ability of LY294002 to suppress NLRP3-mediated inflammation, reinforcing the idea that a signaling pathway involving PI3K, GLCCI1, and NLRP3 may shape macrophage function in the context of asthma.

In conclusion, our findings indicate that OVA reduces GLCCI1 levels in macrophages by activating the PI3K pathway. This process activates the NLRP3 inflammasome, which induces IL-1β release. These observations imply that GLCCI1 plays a pivotal role in the interplay between PI3K and NLRP3 in asthma development. Considering the strong ability of activated macrophages to secrete inflammatory cytokines like IL-1β, using PI3K inhibitors could potentially reduce asthma symptoms by boosting the protective effects of GLCCI1 in these cells.

## Funding

This research was supported by National Natural Science Foundation of China (Nos. 82270033; 81873407), and Natural Science Foundation of Hunan province (No. 2022JJ30924).

## Declaration of competing interest

The authors declare that they have no known competing financial interests or personal relationships that could have appeared to influence the work reported in this paper.

## Appendix Supplementary materials

Image, application 1
