# Supplementary material for: Role of GLCCI1 in inhibiting PI3K-induced NLRP3 inflammasome activation in asthma
Source: Chin Med J Pulm Crit Care Med. 2024 Dec 17;2(4):279–88. doi: 10.1016/j.pccm.2024.11.007 (PMC11742361; doi:10.1016/j.pccm.2024.11.007)
Supplement: Supplementary file 1 [file mmc1.docx]

Supplementary Fig. 1. Western blot analysis of GLCCI1 in bone marrow-derived macrophages from wild type mice and GLCCI1 knock out mice.


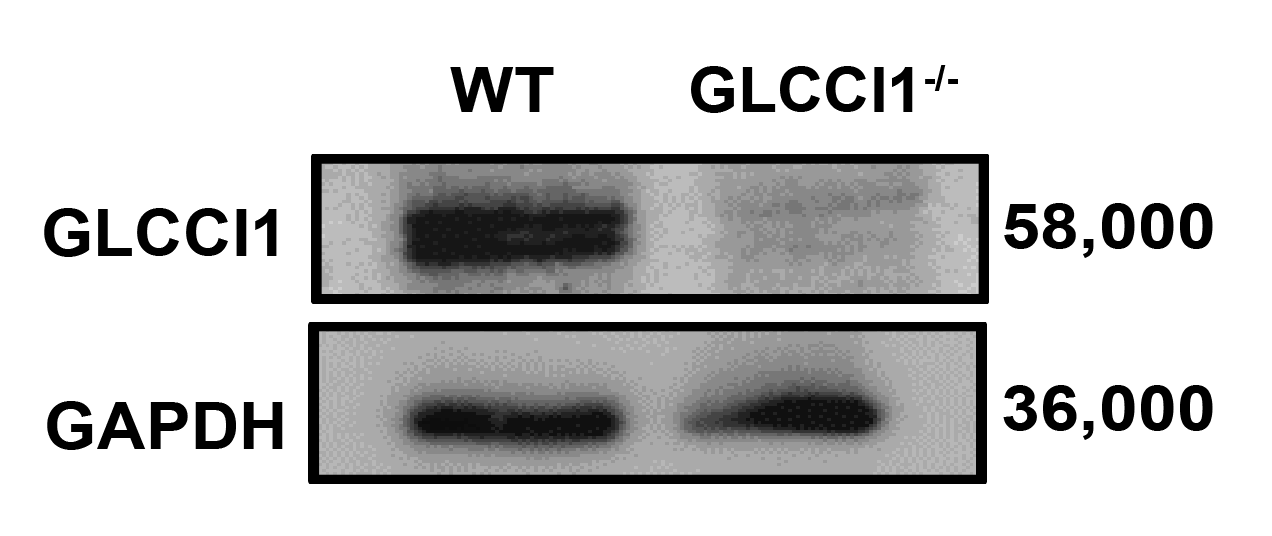


Supplementary Table 1: Primer Sequences used for RT-PCR.

| Gene | Primer sequences |
| --- | --- |
| Mouse-GAPDH（Forward） | GGTTGTCTCCTGCGACTTCA |
| Mouse-GAPDH（Reverse） | TGGTCCAGGGTTTCTTACTCC |
| Mouse-GLCCI1（Forward） | AGGCGAACCTCTTCTCTGGA |
| Mouse-GLCCI1（Reverse） | GTGAACATGAGGGTCCCGTG |
| Mouse-NLRP3（Forward） | ATTACCCGCCCGAGAAAGG |
| Mouse-NLRP3（Reverse） | TCGCAGCAAAGATCCACACAG |
| Mouse-ASC（Forward） | CTTGTCAGGGGATGAACTCAAAA |
| Mouse-ASC（Reverse） | GCCATACGACTCCAGATAGTAGC |
| Mouse-IL-1β（Forward） | GCAACTGTTCCTGAACTCAACT |
| Mouse-IL-1β（Reverse） | ATCTTTTGGGGTCCGTCAACT |
| Mouse-IL-18（Forward） | GACTCTTGCGTCAACTTCAAGG |
| Mouse-IL-18（Reverse） | CAGGCTGTCTTTTGTCAACGA |
| Mouse-Caspase-1（Forward） | ACAAGGCACGGGACCTATG |
| Mouse-Caspase-1（Reverse） | TCCCAGTCAGTCCTGGAAATG |
| Mouse-IL-4（Forward） | GGTCTCAACCCCCAGCTAGT |
| Mouse-IL-4（Reverse） | GCCGATGATCTCTCTCAAGTGAT |
| Mouse-IL-5（Forward） | CTCTGTTGACAAGCAATGAGACG |
| Mouse-IL-5（Reverse） | TCTTCAGTATGTCTAGCCCCTG |
| Mouse-IL-13（Forward） | CCTGGCTCTTGCTTGCCTT |
| Mouse-IL-13（Reverse） | GGTCTTGTGTGATGTTGCTCA |
| Mouse-TNFα（Forward） | CCCTCACACTCAGATCATCTTCT |
| Mouse-TNFα（Reverse） | GCTACGACGTGGGCTACAG |
| Human-GAPDH（Forward） | GGTGAAGGTCGGAGTCAACG |
| Human-GAPDH（Reverse） | CAAAGTTGTCATGGATGACC |
| Human-GLCCI1（Forward） | ACTCGCAGCATTGACACTCA |
| Human-GLCCI1（Reverse） | ATGAGAGCTGCTCAACGGTC |
| Human-NLRP3（Forward） | ATGCCAGGAAGACAGCATTG |
| Human-NLRP3（Reverse） | TCATCGAAGCCGTCCATGAG |
| Human-Caspase-1（Forward） | TTTCCGCAAGGTTCGATTTTCA |
| Human-Caspase-1（Reverse） | GGCATCTGCGCTCTACCATC |
| Human-ASC（Forward） | AACCCAAGCAAGATGCGGAAG |
| Human-ASC（Reverse） | TTAGGGCCTGGAGGAGCAAG |
| Human-IL-1β（Forward） | ATGATGGCTTATTACAGTGGCAA |
| Human-IL-1β（Reverse） | GTCGGAGATTCGTAGCTGGA |
| Human-IL-18（Forward） | TCTTCATTGACCAAGGAAATCGG |
| Human-IL-18（Reverse） | TCCGGGGTGCATTATCTCTAC |
| Human-PI3Kα（Forward） | CCACGACCATCATCAGGTGAA |
| Human-PI3Kα（Reverse） | CCTCACGGAGGCATTCTAAAGT |
| Human-PI3Kβ（Forward） | TATTTGGACTTTGCGACAAGACT |
| Human-PI3Kβ（Reverse） | TCGAACGTACTGGTCTGGATAG |
| Human-PI3Kγ（Forward） | GGCGAAACGCCCATCAAAAA |
| Human-PI3Kγ（Reverse） | GACTCCCGTGCAGTCATCC |
| Human-PI3Kδ（Forward） | AAGGAGGAGAATCAGAGCGTT |
| Human-PI3Kδ（Reverse） | GAAGAGCGGCTCATACTGGG |

RT–PCR: Reverse transcription–polymerase chain reaction.
